# Newer investigations and management guidelines in uveitis

**Published:** 2010

**Authors:** Rajesh Sinha, Prashant Naithani, Satpal Garg

## Introduction

Intraocular inflammation, or uveitis, incorporates a diverse group of infectious and immune-mediated disorders and is often associated with life-threatening systemic disease and vision-threatening ocular complications. In addition, some conditions masquerade as uveitis posing a diagnostic challenge. Correct set of investigations may aid in timely and accurate diagnosis and treatment of both ocular and any systemic conditions associated, thus decreasing morbidity and mortality. We have made an attempt to review the important articles related to investigations in uveitis published in the indexed journals in recent times.

### Investigations in Anterior Uveitis

Approximately 26% of anterior uveitis is associated with a systemic disease; therefore a complete and thorough evaluation of such patients is warranted.

**Forooghian *et al*.** *(Can J Ophthalmol. 2006;41(5):576-83*) reported results from the Canadian National Uveitis Survey (CNUS), a cross-sectional survey identifying investigation patterns for anterior uveitis in Canada. The authors made evidence-based recommendations for appropriate tests in four commonly encountered settings:

1. Non-granulomatous anterior uveitis in an adult: Human leukocyte antigen (HLA) B-27; if recurrent or chronic: Chest X-ray, venereal disease research laboratory (VDRL) and fluorescent treponemal antibody absorption (FTA-ABS) tests.
2. Granulomatous anterior uveitis in an adult: Chest X-ray, Purified protein derivative (PPD) tuberculin testing, serum angiotensin converting enzyme (ACE) levels, VDRL and FTA-ABS tests.
3. Granulomatous anterior uveitis, suspected sarcoidosis, in an adult or child: Chest X-ray, serum ACE levels; if clinical suspicion high despite these tests normal: High-resolution computerized tomography (CT) (HRCT) scan of the chest or whole body gallium scan with or without biopsy.
4. Anterior uveitis in a child: Anti-nuclear antibody (ANA), HLA B-27.

**Noble *et al*.** *(Can J Ophthalmol 2008;43(6):652-7*) analyzed the cost-effectiveness of investigations of anterior uveitis ordered by ophthalmologists with respect to those recommended by the CNUS. The authors found that up to $75 per patient was spent as additional cost in the investigation of anterior uveitis. Certain commonly conducted tests like complete blood counts (CBC) [ordered in over 65% of cases] and erythrocyte sedimentation rate (ESR) were found to be too nonspecific and of no use to the preliminary diagnosis of anterior uveitis. Similarly, rheumatoid factor (RF), though more frequently ordered, had a lesser positivity rate than ANA if juvenile rheumatoid arthritis was the cause. Elevated serum and urine calcium levels too, have little diagnostic value in sarcoidosis and should, according to authors, not be ordered.

**Nussenblatt RN** in an editorial entitled ‘Investigation of anterior uveitis’ *(Can J Ophthalmol. 2008;43(6):630-3*), commented that some cases of anterior uveitis (like herpetic uveitis) could be diagnosed with the aid of clinical signs and symptoms (unilateral involvement, previous or concurrent keratitis, absent corneal sensations and iris atrophy) alone. The author also suggested that a single episode of mild unilateral uveitis does not warrant investigations. Tests should be conducted only if the disease is severe, recurrent, bilateral or chronic (> three months).

## Uveitis in Children

Children constitute about 5-10% of all new uveitis patients. Pediatric uveitis deserves special consideration for reasons that include relatively poor prognosis, unique systemic associations, and various age-related treatment considerations.

**BenEzra *et al*.** (*Br J Ophthalmol. 2005; 89(4): 444–448*) reviewed 276 (33.1%) patients 18 years or younger out of a total of 821 patients seen over ten years in a uveitis clinic in Israel. Complete blood count, ESR, and C reactive protein (CRP) tests were performed in all cases. A quarter of the cases were termed ‘idiopathic’ due to lack of a diagnostic end point. Juvenile idiopathic arthritis (JIA) was the next most common cause (14.9%) of uveitis in the study group although the authors did not comment about the sensitivity or specificity of the ANA and RF levels used in the diagnosis.

**Narayana *et al*.** (*Indian J Ophthalmol 2003;51:129-32*) investigated 31 children (6.29%) out of a total of 493 uveitic patients in a tertiary eye care centre in south India. The incidence of anterior, intermediate and posterior uveitis was equal in this pediatric cohort. Besides routine investigations like CBC, ESR and urinalysis, other investigations were ordered depending on the differential diagnoses. For example, in the case of anterior uveitis with a history suggestive of arthritis, ANA, RF, ESR and rheumatologist consultation were obtained. In the case of granulomatous anterior uveitis and intermediate uveitis, chest X-ray, PPD tuberculin test and serum ACE were obtained to rule out tuberculosis and sarcoidosis. Juvenile rheumatoid arthritis was diagnosed in four (12.9%) patients and sarcoid panuveitis in two (6.5%) patients. The most common diagnosis was pars planitis (29%) followed by idiopathic anterior uveitis (16.1%).

**Sudershan *et al*.** (*Indian J Ophthalmol 2007;55:199-202*) evaluated 40 patients of diagnosed JIA-associated uveitis between 1998 and 2004. The male:female ratio was 1.3:1 with 85% of cases being pauciarticular JRA. Raised ESR was seen in 29 patients. The RF was found positive in two patients (5%). The ANA was done in 12 patients and was positive only in two (16.6%) patients. With evidence of a similar study performed by **Aggarwal *et al*.** *(Indian J Pediatr. 1996;63(3):301-4*) who did not find a single case of an ANA-positive JIA patient with anterior uveitis, they commented that the spectrum of JIA in India probably differed from that seen in the West.

### Ocular Tuberculosis

The presentation of ocular tuberculosis (TB) can be highly varied and a high index of suspicion is required to prevent irreversible visual consequences. Although PPD tuberculin test and chest radiographs have been the gold standards in diagnosing TB, recent tests like the QuantiFERON-TB assay have come into the market as alternatives to diagnosing latent TB in patients with granulomatous uveitis. In face of growing reports of multidrug-resistant TB, it is worthwhile to follow and evaluate developments in every method of detection of the disease.

**Singh *et al*.** (*Indian J Ophthalmol 2004;52:121*) in a retrospective analysis of 1233 uveitic patients seen in a tertiary care centre in north India from 1996 to 2001, diagnosed TB as the most common causative agent (125 patients). The diagnosis of intraocular TB was made if the patient fulfilled criterion A with B or C: (A) Clinical suspicion of disease where any two of the following features were present:

- Granulomatous anterior uveitis, ii) Active periphlebitis, iii) Neuroretinitis, iv) Retinochorioditis, v) Subretinal granuloma/ abscess. (B) Corroborative evidence of disease (any of the two): i) PPD test > 20 mm/ Necrosis
- Positive X-ray chest iii) Aqueous or vitreous tap positive for *Mycobacterium tuberculosis* by polymerase chain reaction (PCR) iv) Sputum positive for acid-fast bacilli on smear, culture or both. v) Histopathological evidence of TB from cervical or parahilar lymph nodes. (C) Response to the antitubercular treatment.

### QuantiFERON-TB test

Approved by the US Food and Drug Administration in 2002, the QuantiFERON-TB measures and compares the *in vitro* gamma interferon levels secreted by the T-cells in the patient's blood in response to ESAT-6 and CFP-10 mycobacterial antigens. Since the initial commercial kit showed significant false-positive results due to cross-reactions with Bacillus calmete guarene (BCG) antigens in immunized patients, in 2005 a newer version, the QuantiFERON-TB Gold test was introduced that has more MTB-specific antigens, thereby increasing the specificity of the test in detecting latent TB infection.

**Kurup *et al*.** *(Can J Ophthalmol. 2006;41(6):737-40*) compared the PPD skin response with QuantiFERON-TB test in 12 consecutive US-born and non-US-born patients with granulomatous uveitis. The QuantiFERON-TB test did not show any intrinsic merit over PPD testing in the selected patients. The authors concluded that the confounding effect of BCG vaccination rendered interpretation of both tests difficult and suggested that second-generation QuantiFERON-TB tests that are now available may hold promise for use in the uveitis clinic and should be formally evaluated.

**Cordero-Coma *et al*.** *(Eye 2009 Mar 20. [Epub ahead of print]*) conducted a prospective, interventional case series by measuring QuantiFERON levels in 31 referred patients with severe idiopathic chronic uveitis or panuveitis and 52 controls. The prevalence of an immune response to *M. tuberculosis* was 15.38% in controls and 32.25% in uveitis patients (*P*=0.07). Two patients were QuantiFERON indeterminate. Eight QuantiFERON-positive and one QuantiFERON-negative uveitis patients were treated for presumed TB-related uveitis. None had been previously diagnosed with TB. After a nine-month tuberculostatic treatment, seven QuantiFERON-positive and one QuantiFERON-negative patient exhibited decreased intraocular inflammation, visual acuity improvement, and no relapses. The authors stated that estimated QuantiFERON sensitivity and specificity were 82 and 100%, respectively and concluded that QuantiFERON was useful for antituberculous treatment (ATT) decision-making in chronic posterior uveitis patients from areas with an intermediate-high prevalence of TB.

In a retrospective study from Germany, **Mackensen *et al*.** *(Am J Ophthalmol. 2008; 146(5):761-6*) compared the rate of QuantiFERON-TB Gold positivity in 21 patients with serpiginous-like choroiditis to a group of 208 healthy hospital employees, another group of 117 healthy hospital workers after TB contact and 45 randomly tested patients with other uveitis forms. Eleven of 21 serpiginous-like choroiditis patients tested positive, of which three were treated with standard anti-tuberculostatic therapy and they improved. The rest were stable at onset of the study. The rate of QuantiFERON positivity in the healthy control groups was 8.7% and 0.9%, and 13% in the other uveitis subsets. The authors concluded that QuantiFERON-TB Gold testing revealed a high number of positive patients, which indicated a tuberculous etiology in the subset, although whether bacterial activity or secondary immunologic processes were the cause, remained speculative.

**Ang *et al*.** *(Ophthalmology 2009;116(7):1391-6*) determined the role of the QuantiFERON-TB Gold assay in 157 patients with suspected TB uveitis seen over an 18-month period at the Singapore National Eye Center (SNEC) uveitis clinic. The authors implemented a novel method of using treatment response to determine the presumed diagnosis of TB to estimate the accuracy of QuantiFERON-TB Gold assay and PPD skin testing. The study concluded that the QuantiFERON-TB Gold assay was not superior to PPD testing in sensitivity for use as a screening test in suspected TB-related uveitis, although it was more specific than PPD testing in identifying MTB infections. The authors also noted that when faced with discordant results, the clinician could choose to treat the patients with ATT if the QuantiFERON-TB Gold test was positive, if clinically indicated.

### Sarcoidosis

Serum ACE levels and chest radiographs have been the most commonly used investigations to diagnose sarcoidosis in the presence of clinical suspicion. However, with the advent of newer imaging modalities like the HRCT chest, Gallium scan and whole body 18-fluorodeoxyglucose (FDG) positron emission tomography-computed radiographic tomography (PET-CT), equivocal cases can be further investigated and a possible diagnosis can be achieved.

**Weinreb *et al*.** (*Invest Ophthalmol Vis Sci 1979;18(12):1285-87*) measured serum ACE levels in 10 patients with chronic granulomatous uveitis with suspected ocular sarcoidosis without evidence of systemic disease and compared it to levels in 10 patients in other forms of uveitis, 10 patients with active sarcoidosis and 25 normal subjects. The authors reported that serum ACE levels were greater than 2 SD above mean in five of the suspected ocular sarcoidosis patients versus one patient in the other uveitis group (that too within 1 SD) and none in normal subjects. Hence the authors were the first to document that the association of an elevated serum ACE with a chronic granulomatous uveitis suggested the diagnosis of ocular sarcoidosis and serum ACE was a useful ancillary test for diagnosing ocular sarcoidosis when confronted with a patient having chronic diffuse granulomatous uveitis.

**Power *et al*.** *(Ophthalmology 1995;102(12):2007-11*) evaluated the role of combined serum ACE activity and whole-body gallium scanning in diagnosing sarcoidosis in patients with features consistent with ocular sarcoidosis but with normal or equivocal chest radiographs. Twenty-two patients with active ocular inflammation and ultimately biopsy-proven sarcoidosis (sarcoid uveitis group) and 70 patients with active uveitis in whom sarcoidosis also was considered but ultimately had another definitive diagnosis (nonsarcoid uveitis) were compared with respect to their serum ACE levels and gallium scans performed in the beginning of their management. All patients in the sarcoid uveitis group had either an elevated ACE level or an abnormal scan and in 16 patients, results of both tests were abnormal. In no patient in the nonsarcoid uveitis group were results of both tests abnormal. The authors reported that the sensitivity of an elevated ACE in diagnosing sarcoidosis was 73% and the specificity was 83%. Using the combination of a positive gallium scan and an elevated ACE, the specificity for diagnosis was 100% and the sensitivity was 73%. Hence they concluded that the combination of serum ACE level and whole-body gallium scan increased the diagnostic specificity without affecting sensitivity in patients with clinically suspicious ocular sarcoidosis who have normal or equivocal chest radiographs.

**Herbort *et al*.** *(Ocul Immunol Inflamm. 2009;17(3):160-9*) reported recommendations of the first International Workshop On Ocular Sarcoidosis (IWOS). The consensus conference identified seven signs in the diagnosis of intraocular sarcoidosis:

1. mutton-fat keratic precipitates (KPs)/small granulomatous KPs and/or iris nodules (Koeppe/Busacca)
2. trabecular meshwork (TM) nodules and/or tent-shaped peripheral anterior synechiae (PAS)
3. vitreous opacities displaying snowballs/strings of pearls
4. multiple chorioretinal peripheral lesions (active and/or atrophic)
5. nodular and/or segmental periphlebitis (± candlewax drippings) and/or retinal macroaneurysm in an inflamed eye
6. optic disc nodule(s)/ granuloma(s) and/or solitary choroidal nodule, and
7. bilaterality.

The laboratory investigations or investigational procedures that were judged to provide value in the diagnosis of ocular sarcoidosis in patients having the above intraocular signs included

1. negative tuberculin skin test in a BCG-vaccinated patient or in a patient having had a positive tuberculin skin test previously,
2. elevated serum ACE levels and/or elevated serum lysozyme,
3. chest X-ray revealing bilateral hilar lymphadenopathy (BHL),
4. abnormal liver enzyme tests, and
5. chest CT scan in patients with a negative chest X-ray result.

Four levels of certainty for the diagnosis of ocular sarcoidosis (diagnostic criteria) were recommended in patients in whom other possible causes of uveitis had been excluded:

1. biopsy-supported diagnosis with a compatible uveitis was labeled as definite ocular sarcoidosis;
2. if biopsy was not done but chest X-ray was positive showing BHL associated with a compatible uveitis, the condition was labeled as presumed ocular sarcoidosis;
3. if biopsy was not done and the chest X-ray did not show BHL but there were three of the above intraocular signs and two positive laboratory tests, the condition was labeled as probable ocular sarcoidosis; and
4. if lung biopsy was done and the result was negative but at least four of the above signs and two positive laboratory investigations were present, the condition was labeled as possible ocular sarcoidosis.

**Clement *et al*.** *(Br J Ophthalmol. 2009 Aug 26. [Epub ahead of print]*) in a retrospective analysis of 50 consecutive patients with uveitis who were referred for chest HRCT because of suspicion of sarcoidosis, found signs of sarcoidosis on HRCT in 10 (20%) patients. The presence of peripheral chorioretinal punched out lesions and posterior synechiae were significantly related to an abnormal HRCT scan.

**Menezo *et al*.** *(Ocul Immunol Inflamm. 2009;17(3):170-8*) reviewed the medical records of 46 patients with a diagnosis of definite and probable neurosarcoidosis supported by laboratory investigations and exclusion of other causes for the neurological symptoms and found that nine patients suffered from anterior uveitis and four had posterior uveitis. The authors concluded that patients with neurological symptoms and associated intraocular inflammation should have a routine workup for sarcoidosis. Investigations should include magnetic resonance imaging (MRI) scan of the brain and orbits and lumbar puncture in selected cases.

In a prospective study conducted by **Chung *et al*.** (*J Chin Med Assoc 2006;69(10):472–477*), 22 uveitis patients suspected to have sarcoidosis along with seven diagnosed systemic sarcoidosis patients underwent bilateral lower fornix 0.3 × 1 cm conjunctival biopsy. Nine of the 22 uveitic patients (40.9%) had a positive conjunctival biopsy. Of the remaining 13 that tested negative, all had sarcoidosis confirmed by lung or mediastinal lymph node biopsy. The authors concluded that conjunctival biopsy may provide a simple and rapid means of distinguishing uveitis due to sarcoidosis and that it should be considered before other more invasive methods of diagnosis.

### 18-fluorodeoxyglucose positron emission tomography/ computed tomography

#### (18-FDG PET/CT)

**Seve *et al*.** *(Ocul Immunol Inflamm. 2009;17(3):179-84*) retrospectively assessed the value of 18-FDG PET in 18 patients with unexplained chronic uveitis suspected to be due to sarcoidosis. All patients had normal chest CT. After review, 10 (53%) whole-body scans were consistent with sarcoidosis. Subsequent mediastinal biopsies proved sarcoidosis in three patients; two patients were considered as presumed sarcoidosis and six as indeterminate sarcoidosis. The authors stated that 18-FDG PET may show focal uptake suggestive of sarcoidosis in patients with unexplained uveitis and could be considered in patients with a high level of suspicion despite a normal chest CT.

**Tannen *et al*.** *(Ocul Immunol Inflamm. 2008;16(1):25-7*) reported a case where combined whole-body 18-FDG PET-CT scanning was used to aid in the diagnosis of a patient with occult sarcoidosis who presented with persistent bilateral panuveitis after cataract surgery and had undergone an extensive negative workup. Results showed extensive mediastinal adenopathy. Biopsy showed a non-caseating granuloma with associated giant cell formation consistent with a diagnosis of sarcoidosis. The authors concluded that FDG PET-CT scanning generated tomographic scans with excellent sensitivity, spatial resolution, and anatomical landmark identification and may be useful in the workup of idiopathic uveitis.

**Shulman *et al*.** *(Ocul Immunol Inflamm. 2009;17(2):95-100*) imaged four systemic sarcoidosis (but not diagnosed ocular sarcoidosis) patients with 18-FDG PET-CT. Two had been treated for conjunctival melanoma and two had been referred for atypical choroidal tumors. In all cases, PET-CT revealed focal systemic lesions with increased uptake (SUV range 1.7-5.9 kg/mL) suggestive of sarcoid granulomas. The authors were of the opinion that the PET-CT aided in differentiating between a metastatic choroidal tumor and uveal sarcoid granuloma and offered a method to assess the presence and distribution of systemic sarcoidosis.

### Indocyanine Green (ICG) angiography in early diagnosis of Vogt-Koyanagi-Harada (VKH) syndrome:

**Bouchenaki *et al*.** *(Klin Monatsbl Augenheilkd. 2000;216(5):290-4*) in their study noted that ICG angiography contributed to rapid diagnosis in 2/3 patients with early disease and concluded that by showing choroidal lesions not seen by the clinical examination or fluorescein angiography, ICG angiography was essential for a correct workup and follow-up of choroidal lesions in VKH.

### Optical coherence tomography (OCT) and fundus fluorescein angiography (FFA):

**Al-Mezaine *et al*.** *(Int Ophthalmol. 2008;28(6):413-23*) evaluated clinical and OCT findings and outcome of treatment in 51 patients (73 eyes) patients with presumed tubercular uveitis. Thirty-one eyes with macular edema were examined at baseline and at follow-up with OCT. Initial visual acuity (VA) of 20/40 or better was significantly associated with central macular thickness (CMT) of 300 microns or less (*P*=0.0065) and diffuse macular edema (DME) (*P*=0.0484). At final follow-up, there was a significant reduction in CMT (*P*<0.001) associated with a significant improvement in VA (*P*=0.0091). The authors concluded that OCT was useful in monitoring the efficacy of treatment in patients with macular edema.

**Markomichelakis *et al*.** *(Ophthalmology 2004;111(5):946-53*) also studied patterns of macular edema in 70 consecutive patients of uveitis. The authors stated that OCT demonstrated three patterns of macular edema in patients with uveitis: DME, cystoid macular edema (CME), and retinal detachment (RD). Eyes with CME had significantly greater retinal thickness measurements than eyes with DME (*P*<0.001). Multivariate analysis revealed that VA was negatively correlated with increased macular thickness, presence of CME, and RD (*P*<0.05). In patients with uveitis with clear media, the morphologic features of macular edema and macular thickness correlated with VA.

**Tsui *et al*.** *(Semin Ophthalmol. 2009;24(1):29-33*) performed a retrospective chart review of six patients of imtermediate uveitis with acquisition and interpretation of ultra wide field fluorescein angiograms. Two eyes had central, large vessel staining; six eyes had peripheral, small vessel staining. The authors concluded that ultra wide field fluorescein angiography is important to detect inflammation early and follow treatment in patients with intermediate uveitis.

### Diagnostic Vitreous Biopsy in patients with Uveitis

**Garweg *et al*.** *(Eur J Ophthalmol. 2006;16(4):588-94*) evaluated the diagnostic yield of vitrectomy specimen analysis in 56 patients with chronic endogenous uveitis in whom extensive systemic workup had not revealed a specific diagnosis (idiopathic) and medical treatment had not resulted in a satisfying clinical situation. The authors found a specific diagnosis in 17.9% patients and excluded a specific diagnosis in 21.4% patients with idiopathic uveitis. In 60.7% the laboratory investigations were inconclusive. The authors concluded that unlike endophthalmitis, the diagnostic yield of vitrectomy specimen analysis had not been improved by routinely applied methods in cases of idiopathic uveitis. This could probably be attributed to insufficient understanding of the underlying pathophysiologic mechanisms.

**Lobo *et al*.** *(Ophthalmology 2003;110(3):595-9*) performed diagnostic vitreous aspiration tap in 53 patients to distinguish between infective, inflammatory, and malignant causes of uveitis. The initial diagnosis of intraocular malignancy or infection was confirmed in 40% of patients. The remaining patients were treated with immunosuppressives for the ocular inflammation and showed clinical improvement over the follow-up period. No complications, except for retinal detachment in one patient were attributed to the procedure. The authors summarized that vitreous aspiration needle tap seemed to be a safe clinical procedure, which had a high success rate in differentiating between infectious, inflammatory, and malignant causes of uveitis.

**Akpek *et al*.** *(Ophthalmology 1999;106(9):1805-10*) retrospectively reviewed vitreous biopsy samples from 26 patients with treatment-resistant or unusual uveitis. The specimens were classified into three groups: “negative,” “suspicious of malignancy,” and “positive” based on the cytologic features, immunomarkers, and flow cytometry. There was 100% concordance between the cytologic reports and the read-out done in a masked fashion at the time of the study. Ten patients were diagnosed with intraocular central nervous system (CNS) lymphoma based on the vitreous cytology and clinical features. The time interval between the initial presentation and vitreous biopsy was one week to two years, with 80% of the patients diagnosed within the first year. Concomitant CNS involvement was seen in six of the diagnosed patients. The authors pointed out that vitreous cytology was a sensitive, reliable, and reproducible method of diagnosing intraocular-CNS lymphoma and a high index of suspicion based on the clinical findings and course of the uveitis was critically important in decision-making for diagnostic vitrectomy.

